# Spin-controlled wavefront shaping with plasmonic chiral geometric metasurfaces

**DOI:** 10.1038/s41377-018-0086-x

**Published:** 2018-10-31

**Authors:** Yang Chen, Xiaodong Yang, Jie Gao

**Affiliations:** Department of Mechanical and Aerospace Engineering, Missouri University of Science and Technology, Rolla, MO 65409 USA

## Abstract

Metasurfaces, as a two-dimensional (2D) version of metamaterials, have drawn considerable attention for their revolutionary capability in manipulating the amplitude, phase, and polarization of light. As one of the most important types of metasurfaces, geometric metasurfaces provide a versatile platform for controlling optical phase distributions due to the geometric nature of the generated phase profile. However, it remains a great challenge to design geometric metasurfaces for realizing spin-switchable functionalities because the generated phase profile with the converted spin is reversed once the handedness of the incident beam is switched. Here, we propose and experimentally demonstrate chiral geometric metasurfaces based on intrinsically chiral plasmonic stepped nanoapertures with a simultaneously high circular dichroism in transmission (CDT) and large cross-polarization ratio (CPR) in transmitted light to exhibit spin-controlled wavefront shaping capabilities. The chiral geometric metasurfaces are constructed by merging two independently designed subarrays of the two enantiomers for the stepped nanoaperture. Under a certain incident handedness, the transmission from one subarray is allowed, while the transmission from the other subarray is strongly prohibited. The merged metasurface then only exhibits the transmitted signal with the phase profile of one subarray, which can be switched by changing the incident handedness. Based on the chiral geometric metasurface, both chiral metasurface holograms and the spin-dependent generation of hybrid-order Poincaré sphere beams are experimentally realized. Our approach promises further applications in spin-controlled metasurface devices for complex beam conversion, image processing, optical trapping, and optical communications.

## All in a spin over chiral metasurfaces

A novel metasurface allows scientists in the US to generate holograms from circular-polarized light beams, showing promise for ultrathin imaging devices. Metasurfaces are nanometer-thin films with nanostructures that enable control over incident light-waves. Jie Gao and co-workers at the Missouri University of Science and Technology constructed a metasurface that transmits different types of light beams depending on their polarization. Their design comprises two sub-arrays on top of one another - one with nanoapertures in a certain chiral (asymmetrical) shape, while the other is based on its mirror image. The resulting metasurface can transmit holograms when exposed to incident light beams that switch between right- and left-handed circular polarization. Under right-handed polarization, only transmission from one sub-array is enabled while the other is blocked, and vice versa when the polarization changes.

## Introduction

Metasurfaces composed of ultrathin metallic or dielectric nanostructures with subwavelength size and spacing^1–4^ that are able to fully control the electromagnetic wavefront have recently been developed for many applications, such as flat optical elements^5–9^, holograms^10–14^, and vortex beam generation^15–19^. Among the various types of metasurfaces, geometric metasurfaces have drawn the greatest attention for their superior capability in optical phase manipulation^20,21^. The geometric phase or Pancharatnam–Berry phase is introduced by rotating the metallic or dielectric nanostructure in the unit cell when the circularly polarized incident beam is converted to the output beam with the opposite handedness. Compared with other types of metasurfaces, geometric metasurfaces can operate over a broad spectrum with generated phase distributions that are robust against fabrication tolerance and material property variations. However, when the incident beam and the converted output beam change their handedness simultaneously, the sign of the geometric phase produced by the metasurface is reversed, which has limited the ability of geometric metasurfaces to implement spin-switchable functionalities^10,20^. Combining the geometric phase with the propagation phase can overcome this problem, but at the cost of losing the broadband and robust phase properties since the shapes of the nanostructures start to influence the generated phase distributions^22^. Several approaches employing an off-axis design have also presented spin-dependent performance, but complicated optical setups and metasurface designs are required, and the phase reversal issue remains unsolved^23,24^. In addition, chiral supercells designed through collective spin-selective destructive or constructive interference have also been realized. However, supercell-based metasurfaces are inherently sophisticated in both design and fabrication, and the pixel size is usually larger than the wavelength^25,26^.

An alternative scheme to realize spin-controllable geometric metasurfaces is to consider chiral nanostructures as unit cells. By constructing two independently designed subarrays of the two enantiomers of chiral nanostructures and then combining the subarrays into one metasurface, spin-controlled wavefront shaping can be enabled. When a circularly polarized wave with a certain handedness, say right-handed circularly polarized (RCP), is incident on the merged metasurface, the transmission from one subarray, say subarray A, is allowed, while the transmission from the other subarray (subarray B) is strongly suppressed due to the unit-cell chirality. Then, the merged metasurface only shows the transmitted signal with the phase distribution of subarray A. Once the incident handedness is switched from RCP to left-handed circularly polarized (LCP), the generated phase profile of subarray A is still reversed, as in ordinary geometric metasurfaces, but the transmission through subarray A is substantially prohibited, and thus, the merged metasurface only exhibits the phase distribution of subarray B in the transmission. However, the main obstacle for this scheme lies in the design of the chiral nanostructure unit cells. Two-dimensional chiral nanostructures are not truly chiral and thus suffer from weak chiroptical responses^27–30^, while three-dimensional (3D) chiral nanostructures are difficult to fabricate with tailored orientation angles^31–34^. Moreover, the low cross-polarization ratio in the transmitted light, as a common issue for geometric metasurfaces, must also be solved.

In this work, we report a new type of chiral geometric metasurface based on plasmonic stepped nanoapertures to demonstrate spin-controlled wavefront shaping functionalities in the near-infrared wavelength range. Both high circular dichroism in transmission and a large cross-polarization ratio in the transmitted light are achieved simultaneously for the unit-cell stepped nanoaperture, which is analyzed based on plasmonic coupling theory and the Jones matrix method. By merging two independently designed subarrays of the two enantiomers of the stepped nanoaperture, the proposed chiral geometric metasurfaces are constructed and successfully applied to realize chiral metasurface holograms and the spin-dependent generation of hybrid-order Poincaré sphere beams, paving the way for future applications in reconfigurable photonic elements, complex beam generation, optical information processing, and advanced chiroptical biosensing.

## Results

**Fig. 1:**
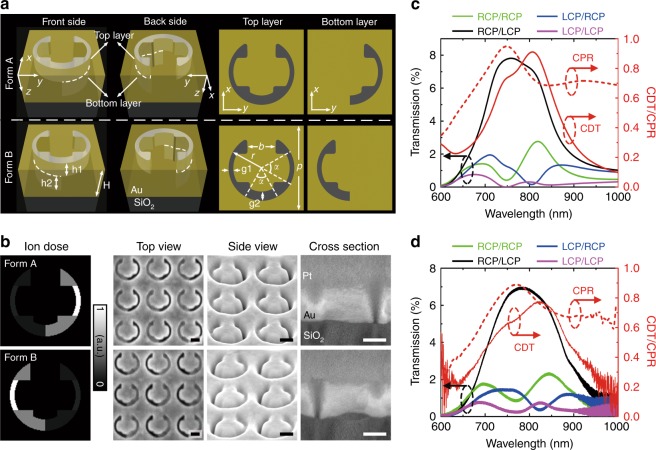
Unit-cell design. **a** Schematic of the chiral stepped nanoaperture in its two enantiomeric forms (Form A and Form B). The thickness of the gold film is *H* = 180 nm. The top and bottom layers of the aperture have thicknesses of *h1* = 80 nm and *h2* = 100 nm, with the cross-sections of the two layers illustrated. The dimension parameters are indicated as *p* = 360 nm, *r* = 140 nm, *b* = 120 nm, *α* = 60°, *g1* = 20 nm, and *g2* = 40 nm. Incoming waves are illuminated vertically onto the top layer and transmitted out of the bottom layer. **b** Normalized ion-dose distributions and SEM images of the stepped nanoapertures fabricated using the grayscale focused ion-beam milling method. Sideview images are captured with a visual angle of 52° to the surface normal. Scale bar: 100 nm. **c** Simulated and **d** measured transmission spectra of the stepped nanoapertures in Form A for different incident/output handedness combinations together with the corresponding CDT and CPR spectra

To simultaneously achieve high circular dichroism and a large cross-polarization ratio in transmitted light, plasmonic stepped nanoapertures etched in an optically thick gold film are proposed as unit cells (Fig. 1a). Distinguished from the unit-cell structures of conventional metasurfaces where nanoantennas or nanoapertures possess a uniform thickness or depth, the proposed plasmonic stepped nanoaperture has two different depths for the left and right halves: one half of the nanoaperture is truncated inside the gold film, while the other half penetrates through the film. Consequently, broken symmetry along the propagation direction is introduced to generate a truly chiral geometry. Depending on whether the left or right half is truncated, the stepped nanoaperture lacking any mirror symmetry exists in two enantiomeric forms (Form A and Form B). The bottom-layer aperture is one half of the top-layer C-shaped structure with a side gap *g1* considerably smaller than the central gap *g2*. Despite its truly 3D structure, the stepped nanoaperture can be easily fabricated with an arbitrary orientation angle using one-step grayscale focused ion-beam milling (Fig. 1b), which is described in the Methods and Supplementary S1.

Transmission spectra for the stepped nanoapertures in Form A are simulated and measured for different incident and output handedness combinations (Fig. 1c, d). A pronounced transmission resonance is observed at ~800 nm under right-handed circularly polarized (RCP) incidence, while the transmission under left-handed circularly polarized (LCP) incidence is substantially suppressed. The circular dichroism in transmission (CDT) is defined as

1 $${{\mathrm{CDT}} = \frac{{\left( {T_{{\mathrm{RCP}}/{\mathrm{RCP}}} + T_{{\mathrm{RCP}}/{\mathrm{LCP}}}} \right) - \left( {T_{{\mathrm{LCP}}/{\mathrm{LCP}}} + T_{{\mathrm{LCP}}/{\mathrm{RCP}}}} \right)}}{{\left( {T_{{\mathrm{RCP}}/{\mathrm{RCP}}} + T_{{\mathrm{RCP}}/{\mathrm{LCP}}}} \right) + \left( {T_{{\mathrm{LCP}}/{\mathrm{LCP}}} + T_{{\mathrm{LCP}}/{\mathrm{RCP}}}} \right)}}}$$

with a simulated CDT resonance of 0.91 at 806 nm and a measured CDT resonance of 0.77 at 820 nm; these values are significantly larger than those for most of the existing chiral metamaterials and metasurfaces, which have a CDT of ~0.4 or even lower^29,30,32^. The wavelength mismatch between the simulated and measured CDT resonances is mainly due to the fabrication tolerance of the focused ion-beam system. In addition, the surface roughness of the gold film and the imperfections of the linear polarizer and quarter wave plate in the experimental setup can also cause such a wavelength shift. Furthermore, most of the transmitted light under RCP incidence possesses converted LCP spin, leading to a large broadband cross-polarization ratio in the transmitted light, *CPR* = *T*_*RCP/LCP*_ /(*T*_*RCP/LCP*_ +*T*_*RCP/RCP*_), with a value of over 0.65 from 700 nm to 1000 nm. The maximum CPR reaches up to 0.89 at 770 nm in experiments, which also indicates the stepped nanoaperture as an excellent half-wave plate element. As a result of the simultaneously enhanced CDT and CPR, the RCP/LCP (incident/output) component carrying the desired geometric phase distribution is remarkably stronger than the other three output components, rendering the stepped nanoapertures suitable for constructing spin-switchable chiral geometric metasurfaces.

**Fig. 2:**
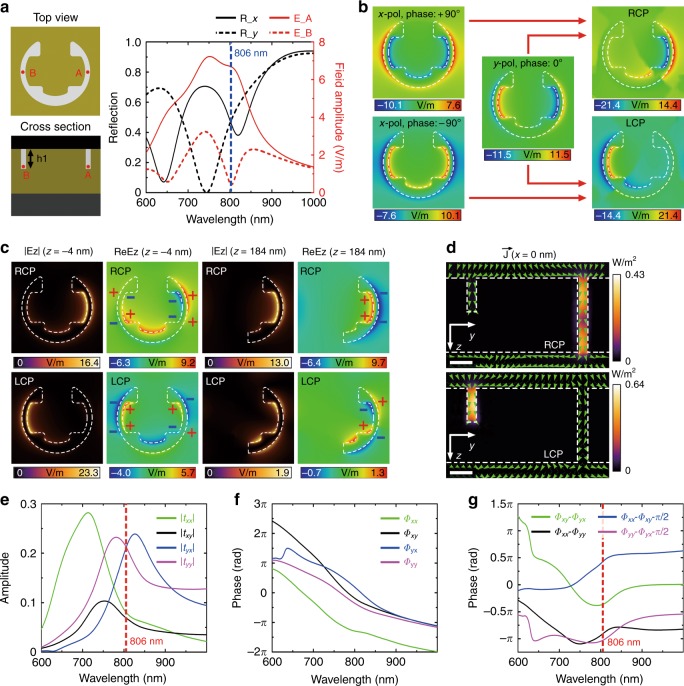
Physical mechanism of the large CDT and CPR. **a** Simulated reflection spectra of the C-shaped groove structure shown in the left panel under *x*-polarized and *y*-polarized illumination (*R_x* and *R_y*) and the respective electric field amplitudes extracted from the two side gaps (*E*_*A*_ and *E*_*B*_) under RCP incidence. The field points A and B are 4 nm above the bottom of the groove structure. The groove structure possesses the same shape as the top-layer structure of the stepped nanoaperture. **b** *E*_*z*_ distributions of the C-shaped groove structure under *x*-polarized and *y*-polarized incidence with a phase delay at 806 nm and the resulting *E*_*z*_ distributions under RCP and LCP illumination. **c** Longitudinal electric field distributions above the top layer (*z* = -4 nm) and below the bottom layer (*z* = 184 nm) of the stepped nanoaperture in Form A at 806 nm, where the absolute value |*E*_*z*_| and the real part *Re*(*E*_*z*_) are plotted. The origin of the *z*-coordinate is set at the top surface. **d** Cross-sectional optical power flow distributions at 806 nm for Form A. The background colors and the field arrows indicate the magnitude and direction, respectively, of the Poynting vector. Scale bar: 50 nm. **e** Amplitude and **f** phase of the Jones transmission matrix of the stepped nanoaperture in Form A under the Cartesian base retrieved from simulations. **g** Calculated phase difference between different components of the transmission matrix

For most chiral metamaterials or metasurfaces, circular dichroism originates from spin-dependent ohmic dissipation or symmetry-breaking substrate effects^30,35,36^. However, the high CDT of the stepped nanoapertures is attributed to the circular dichroic mode coupling process inside the structure. We start from the C-shaped groove structure with an identical depth *h1*, as shown in the left panel of Fig. 2a. The reflection spectra for the C-shaped groove structure under *x*-polarized and *y*-polarized illumination indicate two plasmonic modes with the largest spectral overlap at 806 nm (Fig. 2a). When an *x*-polarized wave is illuminated, the symmetric plasmonic mode is excited inside the structure, where the two electric dipoles located at the two side gaps oscillate in phase with each other (Fig. 2b). However, for *y*-polarized incidence, the antisymmetric plasmonic mode is induced with the two electric dipoles oscillating π out of phase (Fig. 2b). The electric field in the central gap is relatively weaker than that in the side gaps due to the enlarged gap size. When circularly polarized excitation is applied, the two plasmonic modes are simultaneously excited with a relative +π/2 or −π/2 phase delay and interfere with each other. For the RCP case, the interference is constructive in the right arm and destructive in the left arm, which is reversed for the LCP case. Since the two plasmonic modes possess the largest spectral overlap at 806 nm (Fig. 2a), the electric field is highly enhanced in the right (left) arm, while it is extremely weak in the left (right) arm under RCP (LCP) incidence at this wavelength (Fig. 2b). Strong near-field chirality is thus presented for the C-shaped groove structure, even though no chirality is observable in the far field due to the mirror symmetry of the structure.

The stepped nanoaperture proposed here can be regarded as a bilayer structure, where the C-shaped groove structure in the top layer is connected with a half C-shaped aperture in the bottom layer on the right or left for Form A or B. When circularly polarized waves are incident, spin-dependent plasmonic modes are excited inside the top-layer groove structure and then coupled to different modes of the bottom-layer aperture, depending on the field overlap conditions between the different modes at the interface of the two layers. For the RCP case at 806 nm, the electric field is highly enhanced in the right gap area of the top-layer groove structure, exhibiting electric dipole features, as indicated by the *Re*(*E*_*z*_) distributions. This part of the field is further coupled to the superradiant dipole mode of the bottom-layer aperture to generate transmission resonance (Fig. 2c). For the LCP case, the electric field is substantially weaker in the right gap area demonstrating electric quadrupole features, which is then coupled to the subradiant quadrupole mode of the bottom-layer aperture, resulting in low transmission. It is noted that the CDT resonance of the stepped nanoaperture matches well with the near-field chirality resonance of the C-shaped groove structure. As further revealed by the optical power flow distributions (Fig. 2d), the incoming RCP wave is selectively converged into the right gap and transmitted through the gold film, while the incident LCP wave is primarily focused into the left gap and then reflected back by the truncation surface. Detailed chiroptical analysis is provided in Supplementary S2. Our proposed stepped metasurface offers a new design scheme for chiral metasurfaces, whose chirality relies on the spin-dependent mode coupling process between the top layer and the bottom layer, but not on circularly dichroic absorption.

Moreover, the Cartesian Jones transmission matrix ***T***_***cart***_ retrieved from simulations is utilized to analyze the transmission properties of the stepped nanoapertures. By employing the coordinate transformation matrix $${\boldsymbol{\Lambda }} = \frac{1}{{\sqrt 2 }}\left[ {\begin{array}{*{20}{c}} 1 & 1 \\ i & {{\mathrm{ - i}}} \end{array}} \right]$$, the circular Jones transmission matrix ***T***_***circ***_ is calculated as

2 $$T_{{\mathrm{circ}}}{\mathrm{ = }}\left[ {\begin{array}{*{20}{c}} {t_{{\mathrm{RR}}}} & {t_{{\mathrm{RL}}}} \\ {t_{{\mathrm{LR}}}} & {t_{{\mathrm{LL}}}} \end{array}} \right]{\mathrm{ = }}\Lambda ^{{\mathrm{ - 1}}}T_{{\mathrm{cart}}}\Lambda {\mathrm{ = }}\frac{1}{2} \\ \cdot \left[ {\begin{array}{*{20}{c}} {(t_{{\mathrm{xx}}} + t_{{\mathrm{yy}}}){\mathrm{ + i}}(t_{{\mathrm{xy}}} - t_{{\mathrm{yx}}})} & {(t_{{\mathrm{xx}}} - t_{{\mathrm{yy}}}){\mathrm{ - i}}(t_{{\mathrm{xy}}} + t_{{\mathrm{yx}}})} \\ {(t_{{\mathrm{xx}}} - t_{{\mathrm{yy}}}){\mathrm{ + i}}(t_{{\mathrm{xy}}} + t_{{\mathrm{yx}}})} & {(t_{{\mathrm{xx}}} + t_{{\mathrm{yy}}}){\mathrm{ - i}}(t_{{\mathrm{xy}}} - t_{{\mathrm{yx}}})} \end{array}} \right]$$

It is indicated that the components *t*_*yy*_ and *t*_*yx*_, which are significantly larger than the other two components, are almost equivalent in their amplitude at 806 nm (Fig. 2e). At this wavelength, *t*_*yy*_ and *i·t*_*yx*_ are nearly π out of phase with each other (Fig. 2f, g), leading to values for *|t*_*RR*_*|* and *|t*_*LR*_*|* that are much larger than the values for *|t*_*LL*_*|* and *|t*_*RL*_*|*, respectively, and thus a high CDT. Meanwhile, as shown in Fig. 2f, g, *t*_*xx*_ and *t*_*yy*_ are nearly π out of phase with each other from 700 nm to 1000 nm so that |*t*_*xx*_ - *t*_*yy*_| is larger than |*t*_*xx*_ + *t*_*yy*_|, while the phase difference between *t*_*xy*_ and *t*_*yx*_ is below 0.5 π so that |*t*_*xy*_ + *t*_*yx*_| is larger than |*t*_*xy*_ - *t*_*yx*_|, which results in $|t_{{\mathrm{LR}}}|$ being significantly larger than $|t_{{\mathrm{RR}}}|$ and hence the large CPR over a broad spectrum.

**Fig. 3:**
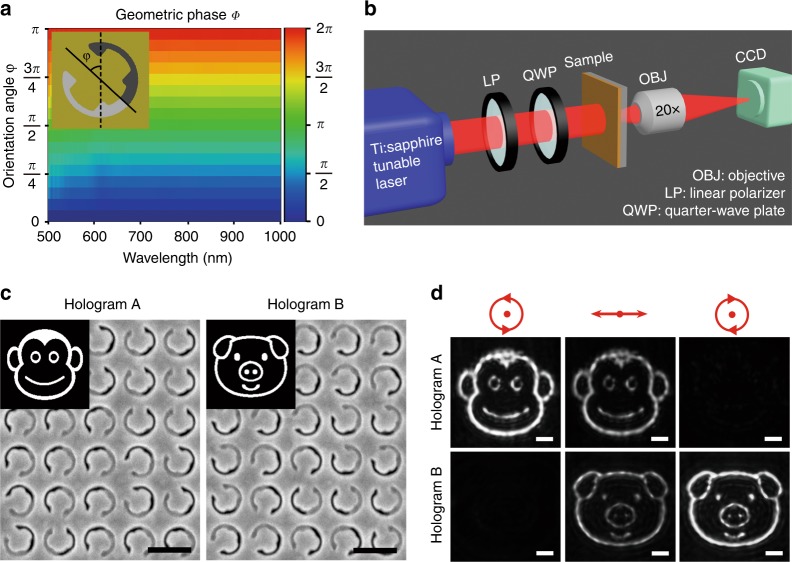
Chiral metasurface hologram based on one enantiomer. **a** Simulated geometric phase delay *Φ* as a function of the orientation angle *φ* of the unit-cell stepped nanoaperture. **b** Illustration of the experimental setups used for capturing holographic images. **c** Target images and partial SEM images of hologram A (Monkey) and hologram B (Pig), constructed with stepped nanoapertures in Form A and Form B, respectively. Scale bar: 500 nm. **d** Reconstructed images for hologram A and hologram B at 820 nm. The incident beams are RCP, linearly polarized and LCP from left to right. Scale bar: 10 μm

As one experimental proof of spin-controllable geometric metasurfaces, chiral holograms are designed to generate distinct holographic images depending on the incident handedness. The Gerchberg–Saxton algorithm is adopted to calculate phase-only CGHs^37^, which are then discretized to 16 phase levels. The relationship *Φ* = *2φ* is robust over a broad spectrum from 650 nm to 1000 nm (Fig. 3a). Benefitting from the high CPR of the unit cells, holographic images are directly captured by an infrared CCD camera without the need for an additional polarization analyzer (Fig. 3b). First, two independent holograms are constructed with the two enantiomers of the stepped nanoapertures (Fig. 3c). The pixel dimension is 360 nm × 360 nm, and the holographic images are reconstructed 27 μm behind the metasurfaces. Owing to the opposite chirality of the two enantiomers, a “monkey” image with high fidelity is observed for hologram A under RCP incidence at 820 nm, while almost no transmission signal is captured for hologram B. When the incident polarization is switched to LCP, the “monkey” image disappears, and the “pig” image is clearly obtained (Fig. 3d). By changing the input spin state, geometric metasurfaces can be switched between ON and OFF states. Here, when the chiral geometric metasurface is switched on, the holography efficiency is measured to be 6.8% at 820 nm, which is comparable with the results of most of existing hologram approaches based on plasmonic metasurfaces in transmission mode^11,14,38–40^. The low efficiency is primarily caused by the large ohmic loss from the plasmonic materials. To solve this problem, dielectric films with lower optical absorption can be employed, such as Si or TiO_2_, to replace the gold film in future work^25,41,42^. The three-layer reflection-type plasmonic metasurfaces can also be utilized to achieve high efficiency^12,43^.

**Fig. 4:**
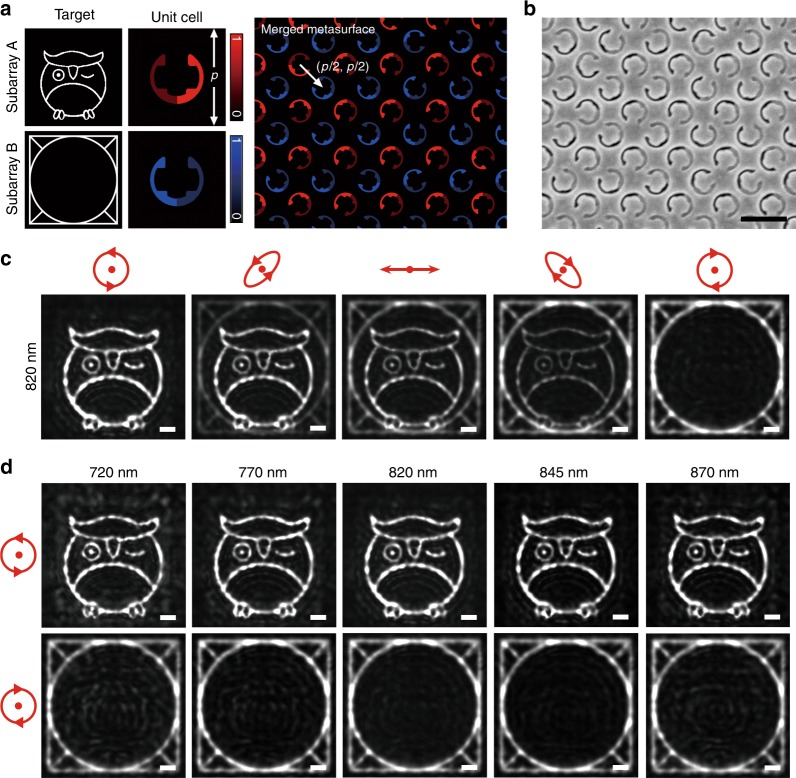
Merged metasurfaces for chiral holograms. **a** Illustration of the chiral metasurface hologram merging subarray A and subarray B to enable spin-controlled wavefront shaping. Subarrays A and B are composed of stepped nanoapertures in Form A and Form B, contributing to the images “owl” and “window”, respectively. **b** Partial SEM image of the merged metasurface corresponding to **a**. Scale bar: 500 nm. **c** Reconstructed images for the chiral metasurface hologram at 820 nm. The polarization states of the incident beam are RCP, right-handed elliptically polarized, linearly polarized, left-handed elliptically polarized, and LCP from left to right. Scale bar: 10 μm. **d** Captured holographic images at different wavelengths of 720 nm, 770 nm, 820 nm, 845 nm, and 870 nm under RCP (top row) and LCP (bottom row) incidence. Scale bar: 10 μm

Furthermore, two subarrays composed of the two enantiomers are interleaved and merged into one combined metasurface (Fig. 4a, b). The unit-cell period *p* is enlarged to 510 nm, and the subarray A is displaced relative to the subarray B with a vector (*p/2*, *p/2*), so that the equivalent unit-cell period remains 360 nm for the merged metasurface. The near-field coupling between neighboring unit cells is weak due to the circular outline of the stepped nanoapertures (Supplementary S3). The hologram resolution defined by *R* = (*λ* × *D*)/(*N* × *P*) is calculated to be 393 nm, where *D* = 44 μm is the distance between the holographic image and the metasurface, *N* = 180 is the pixel number, and *P* = 510 nm is the pixel size. If the combined metasurface is illuminated by a pure RCP beam, only the subarray A is functional for generating an “owl” image, while the transmission from subarray B is almost completely prohibited (Fig. 4c). Once the proportion of the RCP component within the incident beam starts to decrease, the “owl” image becomes darker, and the “window” image gets brighter. When the incident polarization is completely changed to purely LCP, the subarray A is totally disabled, and only the “window” image is obtained. The experimental results suggest that the CDT of the stepped nanoapertures is large enough to avoid mutual disturbance between the two subarrays, and the undesired copolarization transmission is far from deteriorating the holographic image quality due to the high CPR. The entire image evolution process is recoded in Supplementary Movie 1. The broadband response of the chiral metasurface hologram is demonstrated in Fig. 4d. Owing to the geometric nature of the generated phase profiles, clear holographic images are captured at different wavelengths. As the incident wavelength is shifted away from the CDT resonance of 820 nm, the CDT value is continuously decreased, resulting in stronger mutual disturbance between the two subarrays. The quality of the holographic images is thus slightly deteriorated (for more details, see Supplementary S4).

Our approach for realizing chiral geometric metasurfaces is distinct from other spin-switchable metasurface hologram approaches and has several advantages. For multilayer off-axis approaches^23^, deliberate metasurface design is required to diffract holographic images in a certain direction. However, the off-axis design can cause considerable inconvenience and beam misalignment during the optical path building and holographic image capturing process, which is also incompatible with other on-axis optical setups. In addition, a large diffraction angle can cause serious image distortion, which should be carefully compensated. Furthermore, when the operation wavelength is shifted, the diffraction angle is also changed, which is undesired in image acquisition over a broad spectrum. Regarding the hybrid multiplexed hologram^20^, the two holographic images reconstructed under different incident handedness are located at different positions, which requires the adjustment of the objective lens to capture both images. Additionally, we cannot simultaneously acquire the two images by using a linearly polarized incident beam. For supercell approaches^25,26^, each supercell is constructed by a group of nanostructures. Consequently, the design and fabrication of the metasurface is inherently sophisticated. In addition, metasurfaces based on asymmetric spin–orbit interactions can only be applied in the long-wave spectrum due to the large supercell^26^, while binary amplitude-only detour-phase holography suffers from a high noise level and a large waste of pixel resolution^25,44^. Distinguished from all the above spin-controlled approaches based on achiral meta-atoms and realized in indirect ways, our proposed chiral geometric metasurface is constructed by using chiral unit cells to directly enable spin-controlled functionalities. There is no specific requirement for the metasurface design and experimental setup in our approach, which highly extends its flexibility and applicability.

**Fig. 5:**
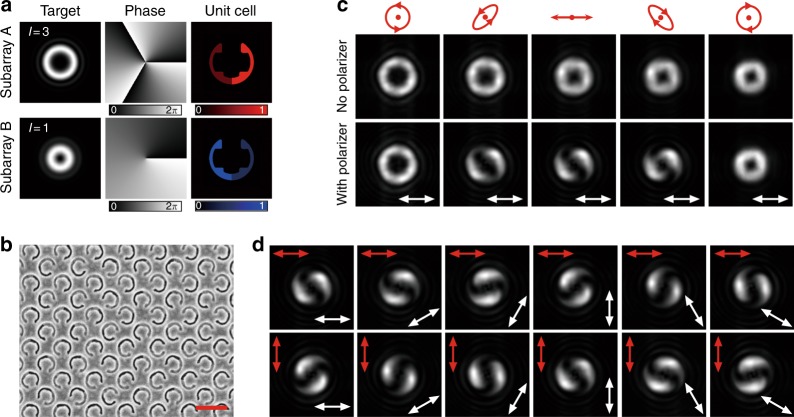
Spin-controlled generation of hybrid-order Poincaré sphere beams. **a** Illustration of the two subarrays to be merged into the combined metasurface. Subarray A and subarray B are responsible for the generation of the OAM modes |*L*, *l* = 3> and |*R*, *l* = 1>, respectively. **b** SEM image of the central part of the combined metasurface. Scale bar: 500 nm. **c** Experimental demonstration of the spin-controlled hybrid superposition of the OAM modes |*L*, *l* = 3> and |*R*, *l* = 1> at 820 nm. *L* or *R* in the bracket indicates a spin state of LCP or RCP, while *l* is the topological charge of the vortex beam corresponding to an OAM of *l*$\hbar$. The polarization states of the incident beam are RCP, right-handed elliptically polarized, linearly polarized, left-handed elliptically polarized, and LCP from left to right. The superimposed mode is investigated by inserting a horizontal linear polarizer before the CCD, as indicated by the white double-headed arrow. **d** Captured intensity profiles of the hybrid-order Poincaré sphere beams under *x*-polarized and *y*-polarized incidence after passing through a rotating linear polarizer. The incident polarization is denoted by the red double-headed arrow, and the transmission axis of the linear polarizer is indicated by the white double-headed arrow

To further demonstrate the versatility of our approach, the spin-controlled hybrid superposition of orbital angular momentum (OAM) states is realized by using the proposed chiral geometric metasurface, which promises wide applications in optical trapping^45^, high-resolution microscopy^46^, and optical communication^47^. In the same way as described before, the metasurface is constructed by merging two subarrays (Fig. 5a, b), which are designed to generate OAM modes of |*L*, *l* = 3> and |*R*, *l* = 1>, respectively. Under RCP incidence, a vortex beam with a |*L*, *l* = 3> mode is produced with a donut-shaped intensity profile around a central dark spot. Once the incident handedness is gradually switched from RCP to LCP, the radius of the dark spot is continuously reduced, suggesting vortex mode evolution from |*L*, *l* = 3> to |*R*, *l* = 1> (Fig. 5c). When the metasurface is illuminated with an elliptically polarized or linearly polarized beam, a so-called hybrid-order Poincaré sphere beam is generated by the superposition of the two modes |*L*, *l* = 3> and |*R*, *l* = 1> with opposite spin states and different absolute values of topological charges^48^, which can be analyzed by a linear polarizer. The polarization state in the beam’s transverse plane is evolved from RCP at the center, through right-handed elliptical polarization and linear polarization, and to left-handed elliptical polarization at the outer edge (see Supplementary S5). In fact, a composite-vortex beam is created after passing through the linear polarizer^49^, where a vortex of *l* = 1 is located at the center and surrounded by two singly charged peripheral vortices, as confirmed by the captured two-lobe shaped intensity profile. For incidence with orthogonal linear polarization, different superimposed OAM modes are produced corresponding to distinct points on the hybrid-order Poincaré sphere, and the obtained two-lobe shaped patterns of the two beams are perpendicular to each other and rotate with the orientation of the linear polarizer (Fig. 5d).

## Discussion

In summary, we have proposed and experimentally demonstrated one unique type of chiral geometric metasurface based on plasmonic stepped nanoapertures to achieve spin-controlled wavefront shaping functionalities, including the realization of both chiral metasurface holograms and the spin-controlled generation of hybrid-order Poincaré sphere beams. The two constituent subarrays of the metasurface are composed of two enantiomers of chiral stepped nanoapertures, so that only one subarray is functional while the other subarray is disabled for a certain incident handedness. The high circular dichroism in transmission and large cross-polarization ratio in the transmitted light of the stepped nanoaperture are attributed to the nanoaperture’s 3D bilayer structure, which opens a new avenue for designing easy-to-fabricate chiral metasurfaces, spin-dependent wave plates, and other special multifunctional metasurfaces. Furthermore, our approach is readily extended to many exciting metasurface-based photonics applications, such as tunable metalenses, 3D image processing, optical trapping, optical routing, structured light conversion and complex vector beam generation, to enable powerful spin-switchable functionalities without the need for complicated metasurface design and optical setup.

## Materials and methods

### Sample fabrication

The stepped nanoapertures are fabricated on a 180-nm-thick gold film with a SiO_2_ substrate using a focused ion-beam system (FEI Helios Nanolab 600, 30 kV, 9.7 pA). Grayscale patterns in bmp format are edited and imported into the system to define the ion-dose distributions in different areas of the stepped nanoapertures. To capture the cross-sectional images of the structure, a layer of platinum is first deposited on the sample using electron-beam-induced deposition. After that, a trench is cut through into the substrate to expose the cross section of the stepped nanoapertures.

### Numerical simulations

All simulations are performed using a commercially available finite element solver package (COMSOL Multiphysics). The refractive index of SiO_2_ is set to 1.45, and the permittivity of gold is extracted from spectroscopic ellipsometry data fitted with a general oscillator model. Periodic boundary conditions are utilized in the *x* and *y* directions, while perfectly matched layers surrounded by scattering boundaries are employed along the *z* direction. All corners of the structure are rounded with a radius of curvature of 10 nm according to the SEM images.

### Experimental setup

In the experimental setup used for holographic image acquisition, a collimated laser beam with tunable wavelength is generated by a femtosecond Ti:sapphire oscillator (Spectra-Physics Tsunami), transmitted through a linear polarizer and a quarter-wave plate, and then illuminated directly onto the metasurface sample to ensure uniform illumination. The reconstructed images collected by a 20× objective are directly captured by an infrared CCD camera. The experimental setup used for capturing the superimposed vortex beam images is similar to that used for holograms, except that the incident beam is slightly focused by a lens with a focal length of 35 mm. A linear polarizer can be inserted before the CCD camera to investigate the superimposed mode.

## Electronic supplementary material

Supplementary Information

Supplementary Movie
