# Supplementary material for: Spin-controlled wavefront shaping with plasmonic chiral geometric metasurfaces
Source: Light Sci Appl. 2018 Oct 31;7:84. doi: 10.1038/s41377-018-0086-x (PMC6207568; doi:10.1038/s41377-018-0086-x)
Supplement: Supplementary file 1 — Supplementary Information [file 41377_2018_86_MOESM1_ESM.docx]

Supplementary Information for

Spin-controlled wavefront shaping with plasmonic chiral geometric metasurfaces

*Yang Chen, Xiaodong Yang*, and Jie Gao**

Department of Mechanical and Aerospace Engineering, Missouri University of Science and Technology, Rolla, MO 65409, USA

*E-mail: (X. Y.) [yangxia@mst.edu](mailto:yangxia@mst.edu), (J. G.) [gaojie@mst.edu](mailto:gaojie@mst.edu).

**S1. Grayscale focused ion beam milling method**


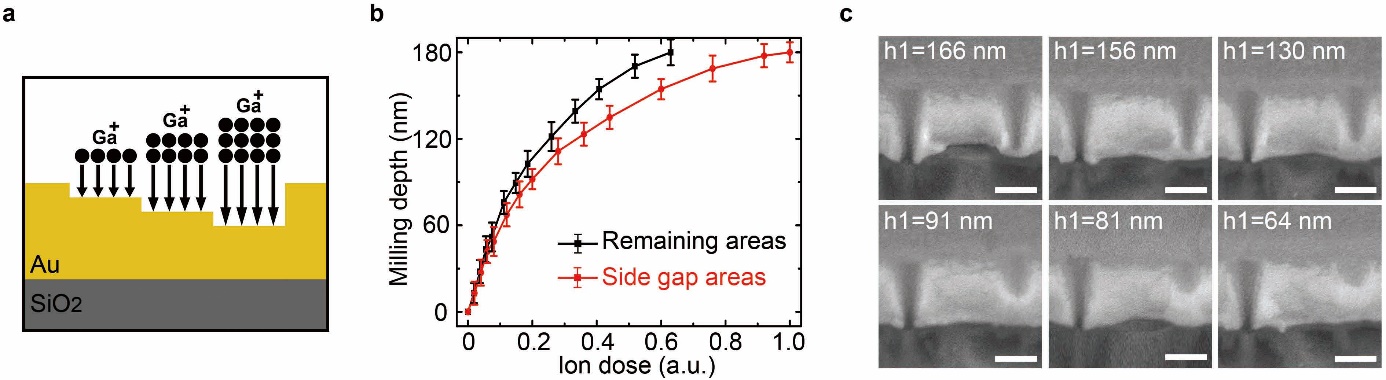


**Fig. S1. a.** Illustration of the grayscale focused ion beam milling method. **b.** Ion dose dependence of the milling depth at the side gap areas and the remaining areas. **c.** Cross-sectional SEM images of the stepped nanoapertures in Form B with different top-layer aperture depth *h1*. Scale bar: 100 nm.

The designed plasmonic stepped nanoapertures are fabricated using grayscale focused ion beam milling method as illustrated in Fig. S1a. Generally, the milling depth follows a linear dependence on the applied ion dose. However, when we are trying to mill a structure with a high depth-to-width ratio, the redeposition effect will substantially reduce the final milling depth[^1^](#_ENREF_1). For the proposed stepped nanoaperture structures, the side gap areas are significantly narrower than the remaining areas, thus more ion dose is required to compensate the redeposition effect there. Based on the experimental data, statistical relationship between the milling depth and the ion dose is established at the side gap areas and the remaining areas, respectively (Fig. S1b). Accordingly, we divide the stepped nanoaperture into four areas in the grayscale milling pattern and arrange different ion doses for different areas (Fig. 1b). Cross-sectional SEM images of the fabricated stepped nanoapertures in Form B with distinct top-layer aperture depths *h1* are shown in Fig. S1c.

**S2. Chiroptical analysis of the stepped nanoaperture**


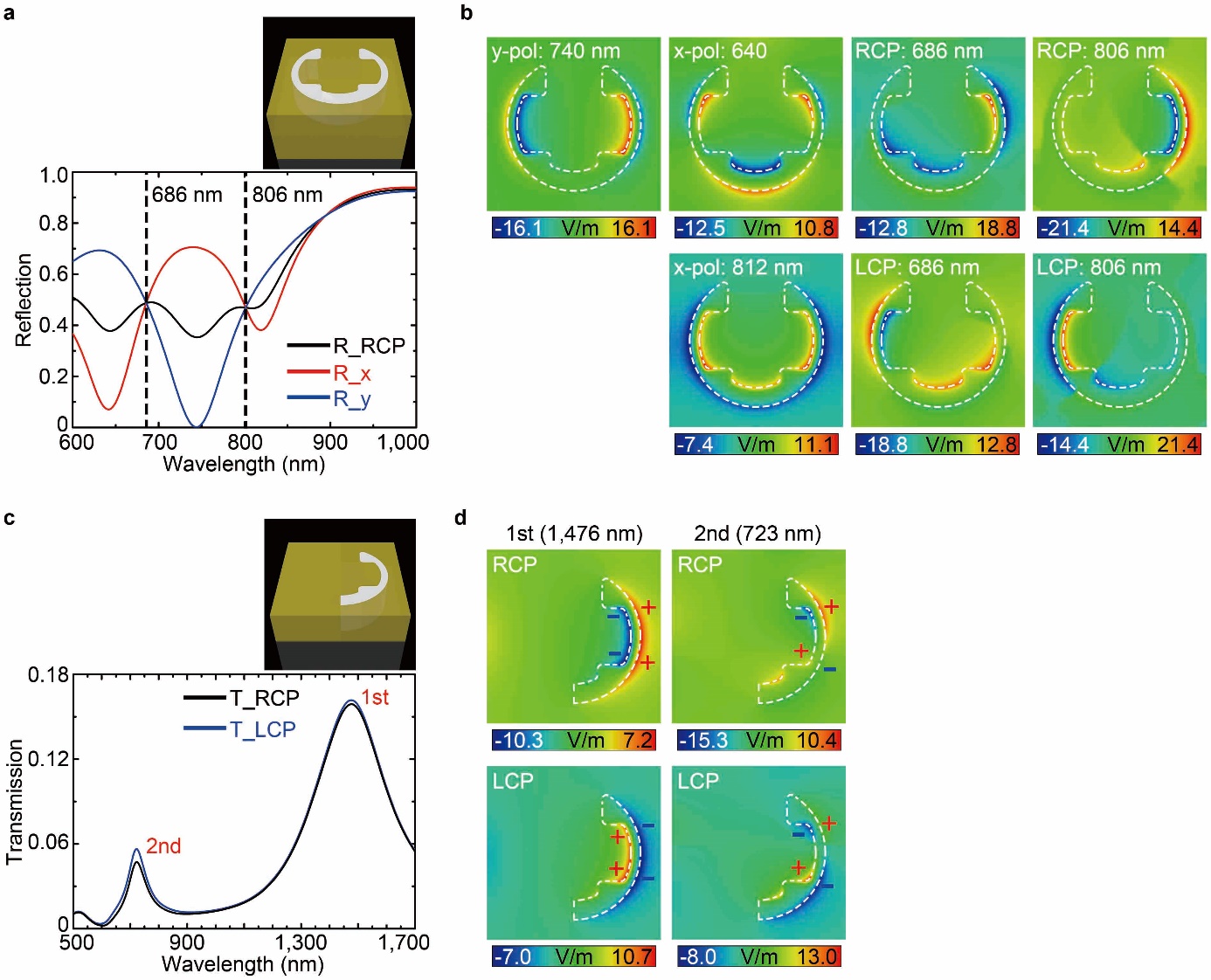


**Fig. S2. a.** Reflection spectra of the C-shaped groove structure under *x*-polarized, *y*-polarized and RCP incidence. **b.** *E_z_* distributions of the C-shaped groove structure for different polarizations and wavelengths. **c.** Transmission spectra of the half C-shaped aperture under RCP and LCP incidence. **d.** *E_z_* distributions of the half C-shaped aperture under RCP and LCP incidence at the first and second resonances.

As described in the manuscript, the high CDT of the stepped nanoaperture is originated from the spin-dependent mode coupling process between the top-layer groove and the bottom-layer aperture. Thus, it is imperative to conduct mode analysis for the two structures, respectively. As shown in Fig. S2a, three reflection resonances are observed for the C-shaped groove structure under RCP incidence, corresponding to the two *x*-polarized modes and one *y*-polarized mode of the structure. For the *y*-polarized mode at 740 nm, two electric dipoles with opposite phase are excited at the two side gaps, indicating it as an antisymmetric mode (Fig. S2b). For the first-order *x*-polarized mode at 812 nm, two electric dipoles with identical phase are induced at the two side gaps, suggesting it as a symmetric mode. The *y*-polarized mode and the first-order *x*-polarized mode possess the largest spectral overlap at 806 nm and interfere with each other under circularly polarized incidence, leading to the electric field at the right gap area significantly stronger (weaker) than that at the left gap area for RCP (LCP) incidence (Fig. S2b). As to the second-order *x*-polarized mode at 640 nm, although symmetric field distributions are also exhibited, the electric field at the central gap is stronger than that at the side gaps. As a result, although the *y*-polarized mode also possesses large spectral overlap with the second-order *x*-polarized mode at 686 nm, the resulting near-field chirality is not as large as that of 806 nm (Fig. S2b), due to the field mismatch between the *y*-polarized mode and the second-order *x*-polarized mode. Thus, the CDT of the stepped nanoaperture at 686 nm is much smaller than that at 806 nm.

Transmission spectra of the half C-shaped aperture under circular polarization basis are simulated as shown in Fig. S2c. Weak circular dichroism is observed because of the substrate with high refractive index and the broken in-plane mirror symmetry. The transmission resonances at 1476 nm and 723 nm are attributed to the fundamental dipole mode and the second-order quadrupole mode respectively (Fig. S2d).

When the C-shaped groove and half C-shaped aperture are connected and coupled to form the stepped nanoaperture in Form A, the dipole-type field on the right side of the top-layer groove is better overlapped with the dipole mode rather than the quadrupole mode of the bottom-layer aperture for the RCP case at 806 nm, and thus the dipole mode is excited in the bottom-layer aperture. Similarly, the quadrupole mode of the bottom-layer aperture is excited for its better mode overlap with the quadrupole-type field on the right side of the top-layer groove for the LCP case. Because the electric field at the top-layer groove is mainly distributed in the right (left) side under RCP (LCP) incidence, the excited dipole mode at the bottom-layer aperture for the RCP case is much stronger than the excited quadrupole mode for the LCP case. Meanwhile, the dipole mode is an intrinsically superradiant ‘bright’ mode, while the quadrupole mode is a subradiant ‘dark’ mode[^2^](#_ENREF_2). As a result of the above two reasons, the RCP transmission is much stronger than the LCP transmission for the stepped nanoaperture in Form A.


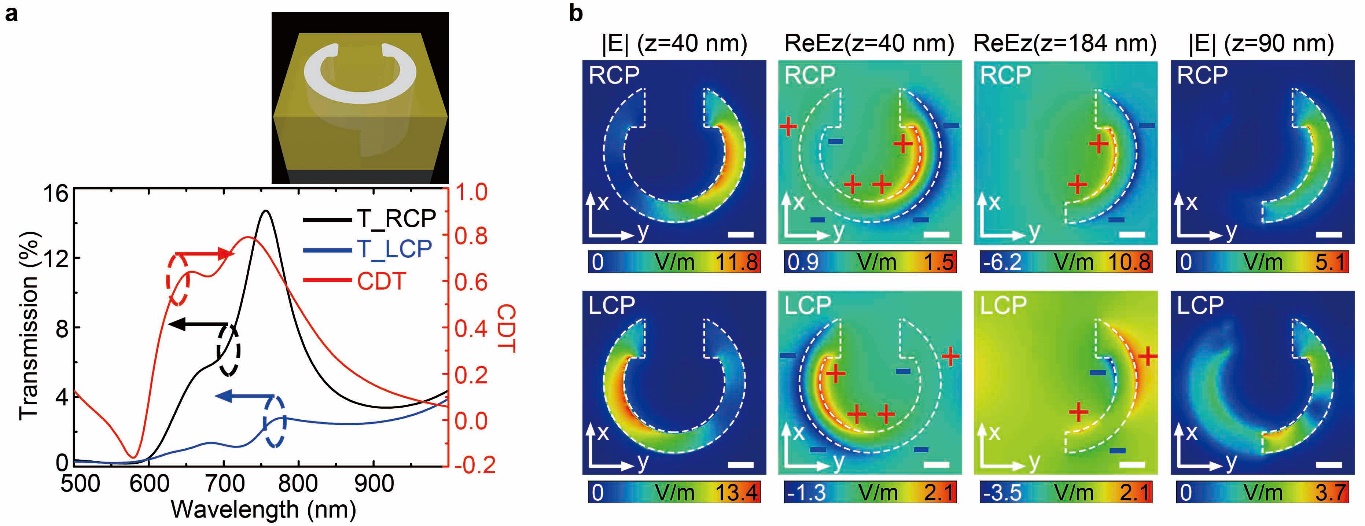


**Fig. S3. a.** Simulated transmission spectra of the stepped uniform-gap nanoaperture in Form A under LCP (T_LCP) and RCP (T_RCP) incidence and the corresponding CDT spectrum. **b.** Electric field distributions of the stepped uniform-gap nanoaperture in Form A at 734 nm. Scale bar: 50 nm.

Next, we are to clarify the importance of having the two side gaps smaller than the central gap for the stepped nanoaperture as mentioned in the manuscript. The chiroptical properties of the stepped nanoaperture with different gap sizes are described in Fig. S2. But for the stepped nanoaperture with a uniform gap size of 40 nm, a transmission peak is presented at 755 nm under RCP incidence, while a transmission dip is observed at 716 nm under LCP incidence with a wavelength shift relative to the transmission peak (Fig. S3a). Meanwhile, the transmission dip is not as low as that of the stepped nanoaperture with different gap sizes. Thus, a CDT resonance of 0.79 is achieved at 734 nm, which is lower than that of the stepped nanoaperture with different gap sizes. Electric field distributions of the stepped uniform-gap nanoaperture at 734 nm are simulated in Fig. S3b. Although the mode field of the top-layer groove is still highly spin-dependent, there are considerable field distributions at the central gap area under LCP incidence. This part of field is then coupled into the bottom-layer aperture to result in considerable transmission under LCP incidence, which is confirmed by the field distributions extracted right below the layer interface (*z* = 90 nm). Accordingly, in order to acquire a large CDT for the stepped nanoaperture, it is important to focus the mode field of the top-layer groove to its side gaps and reduce the field intensity at the central gap, which can be achieved by squeezing the side gap *g1* to be considerably smaller than the central gap *g2*.


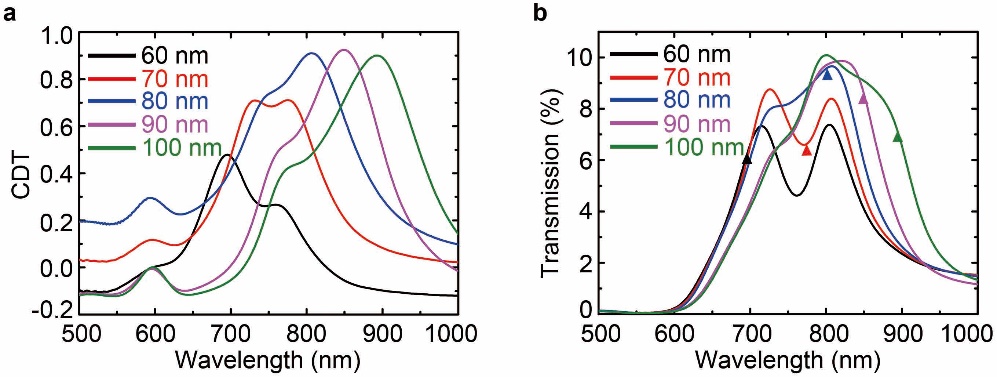


**Fig. S4. a.** CDT spectra of the stepped nanoaperture in Form A for different top-layer depth *h1*. **b.** RCP transmission for different top-layer depth *h1*. The triangles indicate the wavelength positions of CDT resonances for different *h1*.

As another important structural parameter for the stepped nanoaperture, the top-layer depth *h1* is discussed here with the gold film thickness *H* fixed as 180 nm. As shown in Fig. S4a, the CDT resonance for the stepped nanoaperture in Form A is enhanced with increased *h1* until the *h1* value is larger than 80 nm. After that, the CDT resonance is consistently larger than 0.9. Meanwhile, the CDT resonance is continuously redshifted with the increased *h1*. Transmission spectra under RCP incidence for different *h1* are simulated in Fig. S4b, where the locations of CDT resonances are indicated. Thus, the top-layer depth *h1* is set to 80 nm, so that the CDT resonance and the RCP transmission peak are obtained at the same wavelength to achieve both large CDT and high RCP transmission simultaneously.

**S3. Near-field coupling between unit cells**

**
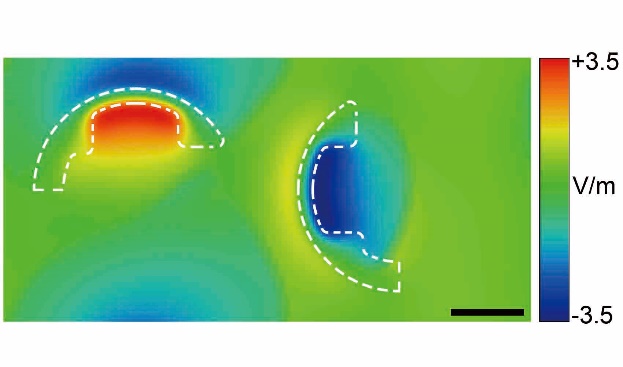
**

**Fig. S5.** Longitudinal field distributions *E_z_* of two neighboring unit cells under RCP incidence, which are extracted 20 nm below the exit plane at 806 nm. Scale bar: 100 nm. The *E_z_* distributions of the two unit cells are out of phase with each other, implying that near-field coupling between the two neighboring unit cells is weak.

**S4. Broadband properties of the chiral meta-holography**

**
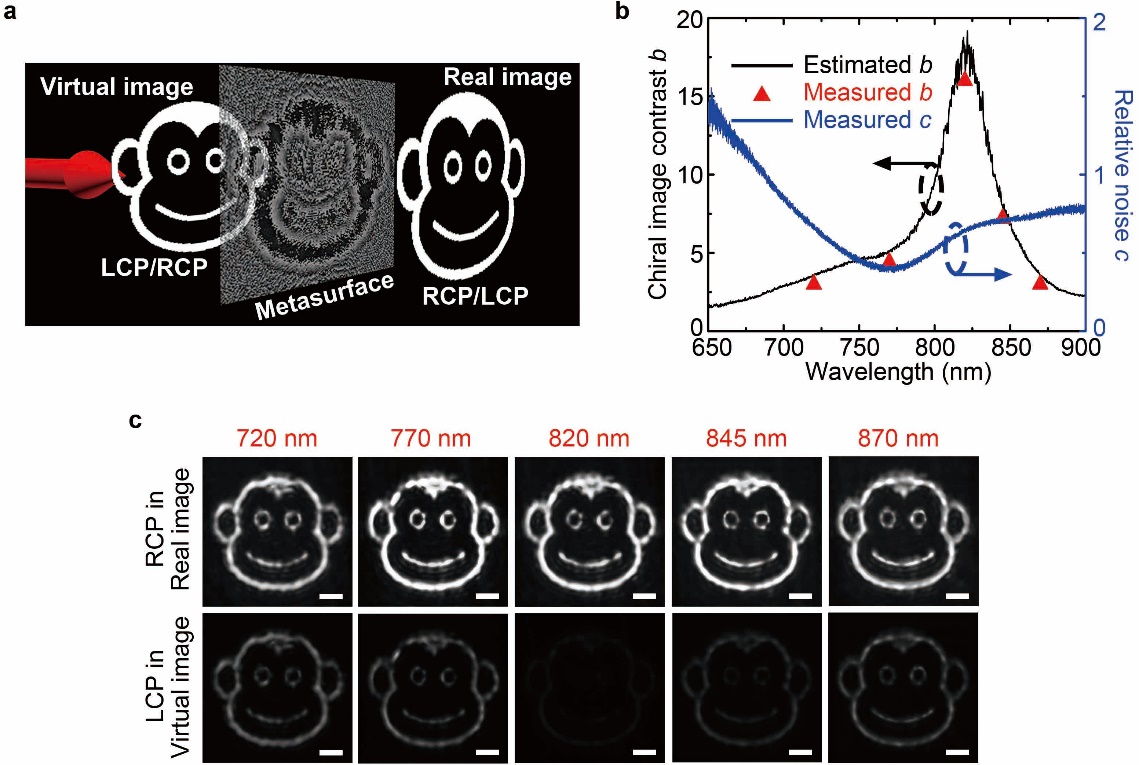
**

**Fig. S6. a.** Illustration of the real image and virtual image generated by the geometric metasurface under different incident/output handedness combinations. **b.** Estimated and measured chiral image contrast *b* together with the measured relative noise *c*. **c.** Captured real images and virtual images for the hologram A at different wavelengths.

For geometric metasurface holograms, if the spin states of the input beam and the converted output beam are switched simultaneously, a virtual image is generated at the opposite side of the metasurface, which is mirror symmetric with the real holographic image relative to the metasurface[^3^](#_ENREF_3)^,^ [^4^](#_ENREF_4) (Fig. S6a). The main task for chiral metasurface holograms is to eliminate such virtual image, which is evaluated by the parameter *chiral image contrast* *b* = *I_real_* / *I_virtual_*, where *I_real_* and *I_virtual_* are total intensities of the captured real image and virtual image.

Among the four different incident/output handedness components of the stepped nanoaperture in Form A (Fig. 1c, d), the RCP/LCP component contributes to the real image reconstruction, while the LCP/RCP component is responsible for the virtual image reconstruction. Thus, the chiral image contrast *b* can be estimated by the ratio *T_RCP/LCP_* / *T_LCP/RCP_*, which is calculated to reach the maximum value of 17.8 at 820 nm. The estimated value matches well with the measured chiral image contrast of 16.2 at this wavelength (Fig. S6b). Such a chiral image contrast is large enough to almost completely prohibit the generation of virtual images as depicted in Fig. S6c. Away from 820 nm, the chiral image contrast is decreased and the virtual image becomes more apparent. Accordingly, the operational wavelength of the chiral metasurface hologram is chosen to be 820 nm in the manuscript. The other two co-polarization components RCP/RCP and LCP/LCP carry no phase information and form an almost uniform background for the holographic images, whose influence can be judged by the *relative noise* *c=* $\sqrt{(\text{T}_{\text{RCP/RCP}}\text{ + }\text{T}_{\text{LCP/LCP}}\text{)}\text{ / }\text{T}_{\text{RCP/LCP}}}$ . As discussed in the reference[^5^](#_ENREF_5), if the relative noise is below 2, the background noise is insignificant for the holographic image, which is well fulfilled by our approach. The captured holographic images also verify that the background noise is weak over a broad spectrum.


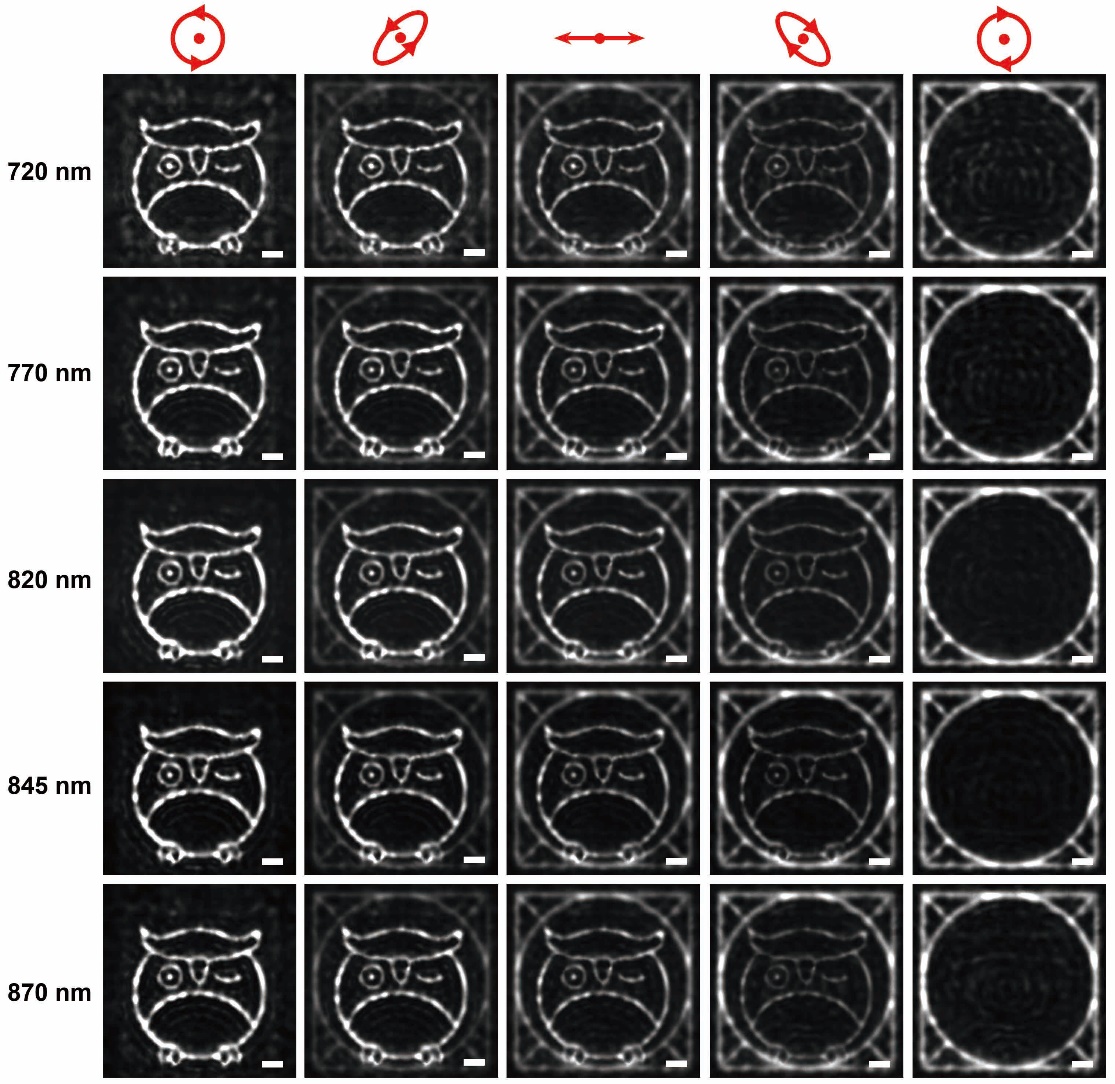


**Fig. S7.** Captured holographic images for the chiral metasurface hologram at different wavelengths of 720 nm, 770 nm, 820 nm, 845 nm and 870 nm from top to bottom. The polarization states of the incident beam are RCP, right-handed elliptically polarized, linearly polarized, left-handed elliptically polarized and LCP from left to right. Scale bar: 10 μm.

Next, the broadband responses of the chiral metasurface hologram are demonstrated. The distance between the holographic image and the metasurface follows an inverse relation with the wavelength. If a RCP beam is illuminated, the LCP transmission produced by the subarray A forms a bright ‘owl’ image after the metasurface, while the LCP transmission from the subarray B produces a virtual ‘window’ image with weak intensity before the metasurface as discussed previously. When the collecting objective is focused after the metasurface to capture the ‘owl’ image, the defocused image of the virtual ‘window’ is also obtained, which is a disturbance for the ‘owl’ image. Such a disturbance can be evaluated by the ratio *T_RCP/LCP_* / *T_LCP/RCP_* of the stepped nanoaperture in Form A, because the LCP/RCP transmission of the enantiomer A is equivalent to the RCP/LCP transmission of the enantiomer B. As a result, the mutual disturbance between the two subarrays should be mostly weakened at 820 nm according to Fig. S6, which is confirmed by the experimental results in Fig. S7. At 720 nm and 870 nm where the ratio *T_RCP/LCP_* / *T_LCP/RCP_* is relatively small, the interference caused by the defocused virtual image becomes obvious leading to slightly low chiral image contrast. Even so, the focused real images are still dominant in the captured images, enabling high-performance chiral holograms over a broad spectrum from 720 nm to 870 nm.

**S5. Polarization analysis of the hybrid-order Poincaré sphere beams**

**
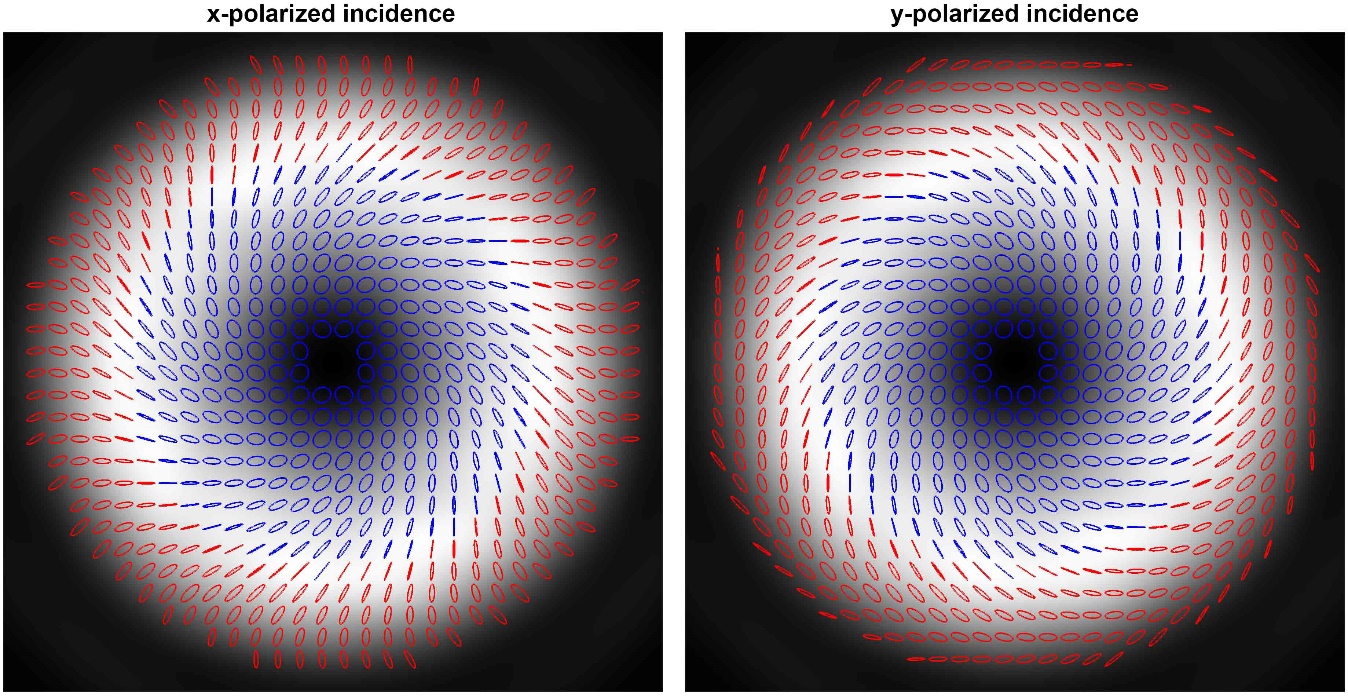
**

**Fig. S8.** Simulated polarization distributions at the transverse plane of the hybrid-order Poincaré sphere beams produced by the merged metasurface in Fig. 4 under *x*-polarized (left) and *y*-polarized (right) incidence. The blue and red colors indicate the right-handedness and left-handedness. Because the radius of the donut-shaped intensity profile of the |*R*, *l* = 1> mode is smaller than that of the |*L*, *l* = 3> mode, the polarization state at the center of the superimposed beams is almost purely RCP. When the position is moving from the center to the outer edge, the polarization state is gradually evolved from RCP through right-handed elliptical polarization and linear polarization and finally to left-handed elliptical polarization. For *x*-polarized and *y*-polarized incidence, different superimposed OAM modes are generated, corresponding to different points on the hybrid-order Poincaré sphere.

**Supplementary References**

1. Melngailis J. Focused ion beam technology and applications. *Journal of Vacuum Science & Technology B: Microelectronics Processing and Phenomena* **5**, 469-495 (1987).

2. Wu C*, et al.* Spectrally selective chiral silicon metasurfaces based on infrared Fano resonances. *Nat Commun* **5**, 3892 (2014).

3. Huang L*, et al.* Three-dimensional optical holography using a plasmonic metasurface. *Nat Commun* **4**, 2808 (2013).

4. Huang L*, et al.* Broadband Hybrid Holographic Multiplexing with Geometric Metasurfaces. *Adv Mater* **27**, 6444-6449 (2015).

5. Huang K*, et al.* Silicon multi-meta-holograms for the broadband visible light. *Laser & Photonics Reviews* **10**, 500-509 (2016).
